# Mitochondrial energy metabolism-related gene signature as a prognostic indicator for pancreatic adenocarcinoma

**DOI:** 10.3389/fphar.2024.1332042

**Published:** 2024-03-20

**Authors:** Yu Ma, Ronghao Tang, Peilin Huang, Danhua Li, Meijian Liao, Shoucui Gao

**Affiliations:** ^1^ Department of Pathology, Xuzhou Medical University, Xuzhou, China; ^2^ School of Medicine, Southeast University, Nanjing, China

**Keywords:** pancreatic adenocarcinoma, mitochondrial energy metabolism, prognostic signature, bioinformatics, immune

## Abstract

**Background:** Pancreatic adenocarcinoma (PAAD) is a highly malignant gastrointestinal tumor and is associated with an unfavorable prognosis worldwide. Considering the effect of mitochondrial metabolism on the prognosis of pancreatic cancer has rarely been investigated, we aimed to establish prognostic gene markers associated with mitochondrial energy metabolism for the prediction of survival probability in patients with PAAD.

**Methods:** Gene expression data were obtained from The Cancer Genome Atlas and Gene Expression Omnibus databases, and the mitochondrial energy metabolism–related genes were obtained from the GeneCards database. Based on mitochondrial energy metabolism score (MMs), differentially expressed MMRGs were established for MMs-high and MMs-low groups using ssGSEA. After the univariate Cox and least absolute and selection operator (LASSO) analyses, a prognostic MMRG signature was used in the multivariate Cox proportional regression model. Survival and immune cell infiltration analyses were performed. In addition, a nomogram based on the risk model was used to predict the survival probability of patients with PAAD. Finally, the expression of key genes was verified using quantitative polymerase chain reaction and immunohistochemical staining. Intro cell experiments were performed to evaluated the proliferation and invasion of pancreatic cancer cells.

**Results:** A prognostic signature was constructed consisting of two mitochondrial energy metabolism–related genes (MMP11, COL10A1). Calibration and receiver operating characteristic (ROC) curves verified the good predictability performance of the risk model for the survival rate of patients with PAAD. Finally, immune-related analysis explained the differences in immune status between the two subgroups based on the risk model. The high-risk score group showed higher estimate, immune, and stromal scores, expression of eight checkpoint genes, and infiltration of M0 macrophages, which might indicate a beneficial response to immunotherapy. The qPCR results confirmed high expression of MMP11 in pancreatic cancer cell lines, and IHC also verified high expression of MMP11 in clinical pancreatic ductal adenocarcinoma tissues. *In vitro* cell experiments also demonstrated the role of MMP11 in cell proliferation and invasion.

**Conclusion:** Our study provides a novel two-prognostic gene signature—based on MMRGs—that accurately predicted the survival of patients with PAAD and could be used for mitochondrial energy metabolism–related therapies in the future.

## 1 Introduction

Pancreatic ductal adenocarcinoma (PDAC) is the most common malignant pancreatic adenocarcinoma (PAAD), and most patients with PDAC are diagnosed with advanced distant metastasis or vascular invasion; only 10%–20% of patients meet the surgical conditions (Baliyan et al., 2018; Huang et al., 2021; Guo et al., 2022). PDAC has a poor prognosis, with a 1-year survival rate of 20% and a 5-year survival rate of <10% (Baliyan et al., 2018; Orth et al., 2019; Siegel et al., 2020). Primary chemotherapy with FOLFIRINOX or nano albumin-bound (nab)-paclitaxel plus gemcitabine provides a modest improvement in survival rates of pancreatic cancer (Conroy et al., 2011; Von Hoff et al., 2013). Therefore, approaches to enhance the prognosis of patients with pancreatic cancer are required urgently.

As Otto Warburg pointed out in the 1920s, solid tumors utilize glucose and generate excess lactate regardless of oxygen availability, and aerobic glycolysis and mitochondrial dysfunction have been proposed as hallmarks of cancer (Warburg, 1956; Hanahan and Weinberg, 2000). Mitochondrial metabolism provides precursors for macromolecules and generates oncometabolites to support cancer proliferation (DeBerardinis and Chandel, 2020; Vasan et al., 2020). Inhibition of mitochondrial metabolism provides new therapeutic strategies for cancer treatment. In recent years, many studies have shown that reprogramming mitochondrial metabolism contributes to the malignant phenotype of PAAD (Faubert et al., 2020). However, the effect of mitochondrial metabolism on the prognosis of pancreatic cancer has rarely been investigated.

In the present study, mitochondrial energy metabolism–related genes (MMRGs) were identified. RNA-sequencing and clinical data were downloaded from The Cancer Genome Atlas (TCGA) (Weinstein et al., 2013) and Gene Expression Omnibus (GEO) databases (Barrett et al., 2013). After univariate Cox regression and least absolute and selection operator (LASSO)–Cox analysis (Cai and van der Laan, 2020), we successfully established a two-gene prognostic signature to construct a risk model that served as an independent prognostic factor for PAAD; we also performed functional enrichment and immune cell distribution analyses based on the risk model. In addition, a 15-hub gene signature related to the mitochondrial energy metabolism score (MMs)—and based on the risk model—was obtained through Gene Ontology (GO) similarity analysis. Finally, by considering both the clinical information and prognostic gene signature, a nomogram (Park, 2018) was developed to predict the individual survival rate in patients with PAAD.

## 2 Methods

### 2.1 Data collection and preprocessing

RNA-sequencing and clinical data of 178 PAAD and 4 normal samples were obtained from TCGA database (https://portal.gdc.cancer.gov/) for analysis. The relative clinical characteristics of the samples were acquired from the UCSC Xena database (http://genome.ucsc.edu) (Goldman et al., 2020). Gene expression was normalized using the “limma” R package (https://www.r-project.org/) (Hänzelmann et al., 2013). GSE62452 (Yang et al., 2016), GSE57495 (Chen et al., 2015), GSE28735 (Zhang et al., 2012), and GSE16515 (Pei et al., 2009) were downloaded from the GEO database (http://ncbi.nlm.nih.gov/geo/) as validation sets. A total of 178 (TCGA), 69 (GSE62452), 63 (GSE57495), 45 (GSE28735), and 36 (GSE16515) patients with PAAD were finally enrolled in the study. Supplementary Table S1 summarizes detailed patient features.

We also obtained 43 MMRGs from the GeneCards database (https://www.genecards.org/) (Stelzer et al., 2016) (“mitochondrial energy metabolism” as keywords, MMRGs with “Protein Coding” and “Relevance score>1” retained), and 188 genes were obtained from the Kyoto Encyclopedia of Genes and Genomes (KEGG) pathway database (https://www.genome.jp/kegg/pathway.html) (Kanehisa and Goto, 2000). A total of 226 MMRGs were identified after removing duplicates.

### 2.2 Single-sample gene set enrichment analysis (ssGSEA)

To quantify the relative abundance of gene sets enriched in a sample, ssGSEA was used to generate the mitochondrial energy metabolism sore (MMs) in each PAAD sample using the “GSVA” R package (Hänzelmann et al., 2013). With reference to the median value of the MMs, patients were classified into MMs-high and MMs-low groups.

### 2.3 Identification of differentially expressed genes (DEGs)

To determine the DEGs based on MMs-high and -low groups, the “limma” R package was employed (https://www.r-project.org/) (Hänzelmann et al., 2013). Genes with a *p*-value <0.05 and log FC > 1.2 were identified as upregulated genes, and those with a *p*-value <0.05 and log FC < −1.2 as downregulated genes.

### 2.4 Weighted co-expression network analysis (WGCNA)

As a biological approach for evaluating gene association patterns among various samples (Zhang and Horvath, 2005), the “WGCNA” R package (Langfelder and Horvath, 2008) was employed to determine the gene modules associated with the PAAD MMs. The key genes were obtained by the intersection of the DEGs and module genes, which were highly related to the MMs.

### 2.5 Construction and validation of the prognosis for the MMRG-related gene signature

Initially, univariate Cox regression analyses were performed to establish the key prognostic MMRGs associated with survival rates of patients with PAAD. We used genes with a *p*-value <0.1 in subsequent studies. Next, LASSO regression was used to select key prognostic genes, and multivariate Cox regression analyses were performed to verify whether these potential prognostic genes were independent prognostic indicators. TCGA nomogram was identified according to the independent prognostic gene signature via the “rms” R package (Park, 2018). Calibration curves were then plotted to validate the predictability of the model for the survival of patients with PAAD. The MMRG-related risk score for each patient with PAAD was calculated by multiplying the relative expression level by regression coefficients. Patients were divided into high- and low-risk score groups based on the median risk score. Risk scores were also calculated using the GEO datasets (GSE28735, GSE57495, and GSE62452) to validate the prognostic gene signature. Receiver operating characteristic (ROC) curves were used to evaluate efficacy in the validation sets.

### 2.6 GO and KEGG analyses according to the high and low MMRG-related risk score groups

Initially, to build DEGs according to high and low MMRG-related risk score groups, genes with *p*-values <0.05 and log FC > 1.2 were identified as upregulated genes, and those with *p*-values <0.05 and log FC < −1.2 were identified as downregulated genes. GO (Yu, 2020) and KEGG pathway (Kanehisa and Goto, 2000) genomes were used to conduct a series of gene functional enrichment analyses. To identify the functions of PAAD prognosis–related genes and relevant molecular mechanisms, the “clusterProfiler” R package (Yu et al., 2012) was used to perform GO and KEGG analyses (*p* < 0.05 and FDR <0.20).

### 2.7 Identification of hub genes

The “GOSemSim” package (Yu et al., 2010) was used to calculate the GO semantic similarity of genes and the geometric mean values of biological processes, molecular functions, and cellular components of DEGs obtained from the Cox multivariate model. The top 15 DEGs with the highest comprehensive scores were considered hub genes in subsequent studies. Finally, the “ggplot” package was used for the visualization of the functional similarity analysis outcomes.

### 2.8 GSEA

To identify molecular and biological differences, GSEA was performed according to KEGG and HALLMARK gene sets from the molecular signature database (https://www.gsea-msigdb.org/gsea/msigdb) (Liberzon et al., 2015) between subgroups with high or low risk scores, as analyzed using the “clusterProfiler” R package (*p* < 0.05 and FDR <0.25).

### 2.9 Interaction analysis of hub genes

The GeneMANIA website (Franz et al., 2018) was used to predict the functionally similar genes among the screened hub genes. We used the GeneMANIA website to illustrate the functionally similar genes among the prognostic hub genes and construct an interaction network.

The ENCORI (https://starbase.sysu.edu.cn/) (Li et al., 2014) and miRDB (Chen and Wang, 2020) databases were used to predict the miRNAs interacting with hub genes. Next, the mRNA–miRNA interaction network was plotted after the mRNA–miRNA data in the ENCORI database intersected with the data with a Target Score >85 in the miRDB database.

CHIPBase (Zhou et al., 2017) (version 3.0) (https://rna.sysu.edu.cn/chipbase/) and hTFtarget databases (Zhang Q. et al., 2020) (http://bioinfo.life.hust.edu.cn/hTFtarget) were used to predict the transcriptional regulation relationship between several million transcription factors (TFs) and hub genes.

### 2.10 Assessment of immune cell infiltration and immune microenvironment

The “ESTIMATE” R package (Yoshihara et al., 2013) was used to evaluate immune infiltration in PAAD samples. The difference in immune cell infiltration between the high- and low-risk score groups of patients was analyzed using the CIBERSORT algorithm (https://cibersortx.stanford.edu/) (Steen et al., 2020). The “ConsensusClusterPlus” R package (Wilkerson and Hayes, 2010) was used for consensus clustering analysis (Brière et al., 2021), which could classify samples into several subtypes according to different omics data sets, to find new subtypes of diseases or conduct comparative analysis of different subtypes (reps = 100, pItem = 0.8, pFeature = 1). In addition, we predicted the immune checkpoint response in the high- and low-risk score groups using the tumor immune dysfunction and exclusion (TIDE) (http://tide.dfci.harvard.edu/) algorithms (Fang et al., 2020; Fu et al., 2020).

### 2.11 Construction and validation of the prediction nomogram

A nomogram was developed to evaluate the mortality rate of patients with PAAD by combining the MMRG-related risk score of the model and clinical data. Prognostic ROC curves were used to assess the efficacy of predicting patient outcomes.

### 2.12 Cell culture and quantitative polymerase chain reaction (qPCR)

A cDNA microarray, which contained seven pancreatic cancer cell lines, and a normal pancreatic duct epithelial cell line (hTERT-HPNE) were obtained from Shanghai Outdo Biotech Company (Shanghai, China). hTERT-HPNE and SW1990 cell lines were maintained in DMEM (Invitrogen, Carlsbad, CA, United States) supplemented with 1% penicillin-streptomycin (Invitrogen) and 10% fetal bovine serum (Invitrogen) at 37°C and 5% CO_2_. RNA was extracted from hTERT-HPNE using a TRIzol kit (Sigma-Aldrich Co., St. Louis, MO, United States) according to the manufacturer’s instructions. The extracted RNA was reverse transcribed into cDNA using a PrimeScript RT kit (Takara Bio, Inc., Dalian, China). We then performed qPCR analyses using an ABI 7500 PCR system (Thermo Fisher Scientific, Waltham, MA, United States) using ChamQ Universal SYBR qPCR Master Mix (Vazyme Biotech Co., Ltd. Nanjing, China). β-actin was used as the internal control. The primer sequences used were as follows: MMP11‐forward, 5′‐CTT​GCT​GTA​TCC​CTG​TTG​TG‐3′; MMP11‐reverse, 5′‐ACC​CCT​CCC​CAT​TTG​ACT​G; β-actin‐ forward, 5′‐GAA​GAG​CTA​CGA​GCT​GCC​TGA‐3′; and β-actin‐reverse, 5′‐CAG​ACA​GCA​CTG​TGT​TGG​CG‐3′.

### 2.13 Immunohistochemistry

MMP11 was detected using PC tissue microarrays obtained from Shanghai Outdo Biotech containing 51 PC tissure spots and 9 peritumoral tissue spots of 56 patients (TMA; HPanA060CS04, Shanghai, China). In brief, TMAs were incubated with MMP11 antibodies (AF0211,1:500, Affinity Biosciences), and then using the EnVisionTM FLEX + Kit (K8002, Dako, Denmark). Subsequently, samples were imaged using Aperio ImageScope (Leica Biosystems, Wetzlar, Germany). The overall score for each section was assessed by multiplying the intensity score by the percentage score of positively stained cells.

### 2.14 Cell transfection

Cells were transfected with synthesized small interfering RNAs (GenePharma Inc, Shanghai, China) targeting MMP11 using Lipofectamine 2000 (Invitrogen) according to the manufacturer’s protocol. Cells were seeded in 6-well plates, and transfection was performed when the cells reached 70%–80% confluence. The siRNA sequences for gene MMP11 were as follows: MMP11-Homo-775: sense (5′-3′) GGG​CGU​UCA​ACA​CCU​AUA​UTT, antisense (5′-3′) AUA​UAG​GUG​UUG​AAC​GCC​CTT; MMP11-Homo-1407: sense (5′-3′) GGA​AGU​UUG​ACC​CUG​UGA​ATT, antisense (5′-3′) UUC​ACA​GGG​UCA​AAC​UUC​CTT; MMP11-Homo-590: sense (5′-3′) GAU​GUC​CAC​UUC​GAC​UAU​GAU, antisense (5′-3′) AUC​AUA​GUC​GAA​GUG​GAC​AUC; MMP11-Homo-870: sense (5′-3′) GAU​AGA​CAC​CAA​UGA​GAU​UGC; antisense (5′-3′) AAU​CUC​AUU​GGU​GUC​UAU​CCC.

### 2.15 CCK-8 assay

Cell lines were seeded in 96-well plates at a density of 3 × 10^3^ cells/well and then cultivated for 0, 24, 48, or 72 h. Twenty-four hours after siRNA transfection, 10 μL of Cell Counting Kit-8 (CCK-8) solution (Dojindo, Tokyo, Japan) was applied to each well. The absorbance was determined at 450 nm with a spectrophotometric plate reader (Multiskan FC, Thermo Fisher Scientific, Waltham, MA, United States). Subsequently, the relative percentage of viable cells was calculated as follows: (OD450 at detection time/OD450 at 0 h) × 100.

### 2.16 Transwell assay

Transwell chambers (8 μm pores; Corning, Corning, NY, United States) were precoated with 60 μL of Matrigel (1:3 mixed with fetal bovine serum [FBS]-free medium; BD Biosciences, Philadelphia, PA, United States). Afterward, 5 × 10^3^ transfected cells in FBS-free medium were inoculated in the upper chambers for 24 h and 700 μL of medium with 20% FBS was placed in the lower chambers to evaluate the invasive capacity of cells. The cells in the bottom chamber were fixed with paraformaldehyde for 30 min before staining with Giemsa for 30 min. Next, the number of cells was counted under a microscope (Nikon Eclipse, Tokyo, Japan).

### 2.17 Statistical analysis

This study employed GraphPad Prism 8.0 (GraphPad Software Inc., La Jolla, CA, United States) and R software (version 4.1.2) (R Foundation for Statistical Computing, Vienna, Austria) for data analysis. The two-sided Student’s t-test and Kruskal–Wallis test were used for statistical analyses, and the Chi-squared test was employed to distinguish various proportions. Kaplan-Meier analysis was used to evaluate the survival of patients in the different groups. Statistical significance was set to *p* < 0.05.

## 3 Results

**FIGURE 1:**
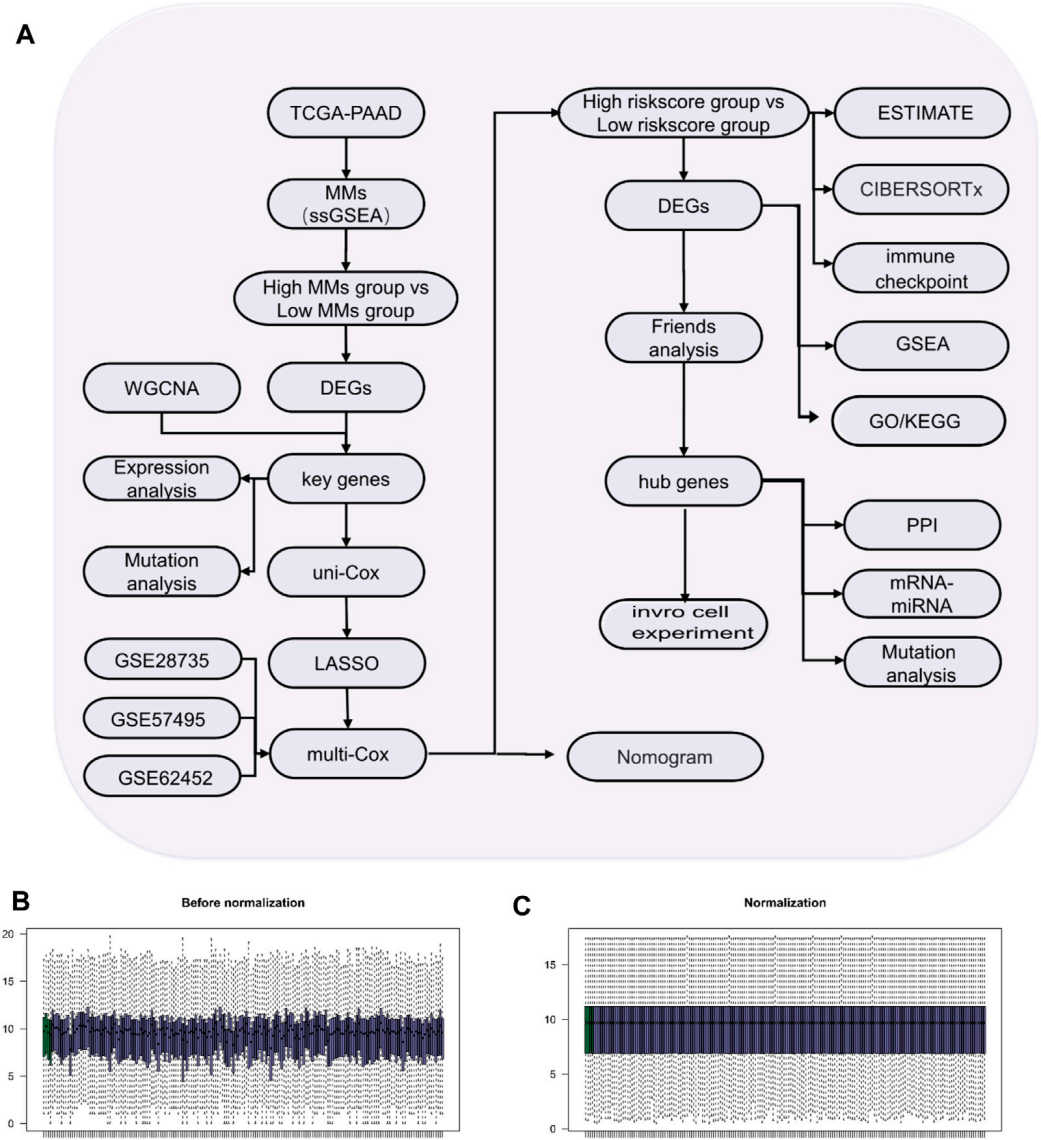
Study process. **(A)** Flow diagram of the study. **(B)** Box-type diagram of TCGA-PAAD dataset before standardization. **(C)** Box diagram of the standardized TCGA-PAAD dataset. The purple sample is the PAAD group (n = 178) and the green sample is the normal group (n = 4).

The flow diagram of the study process is shown in Figure 1A. All data were standardized using the “limma” package in R (Figures 1B,C).

### 3.1 Identification of prognostic MMRGs in TCGA dataset

**FIGURE 2:**
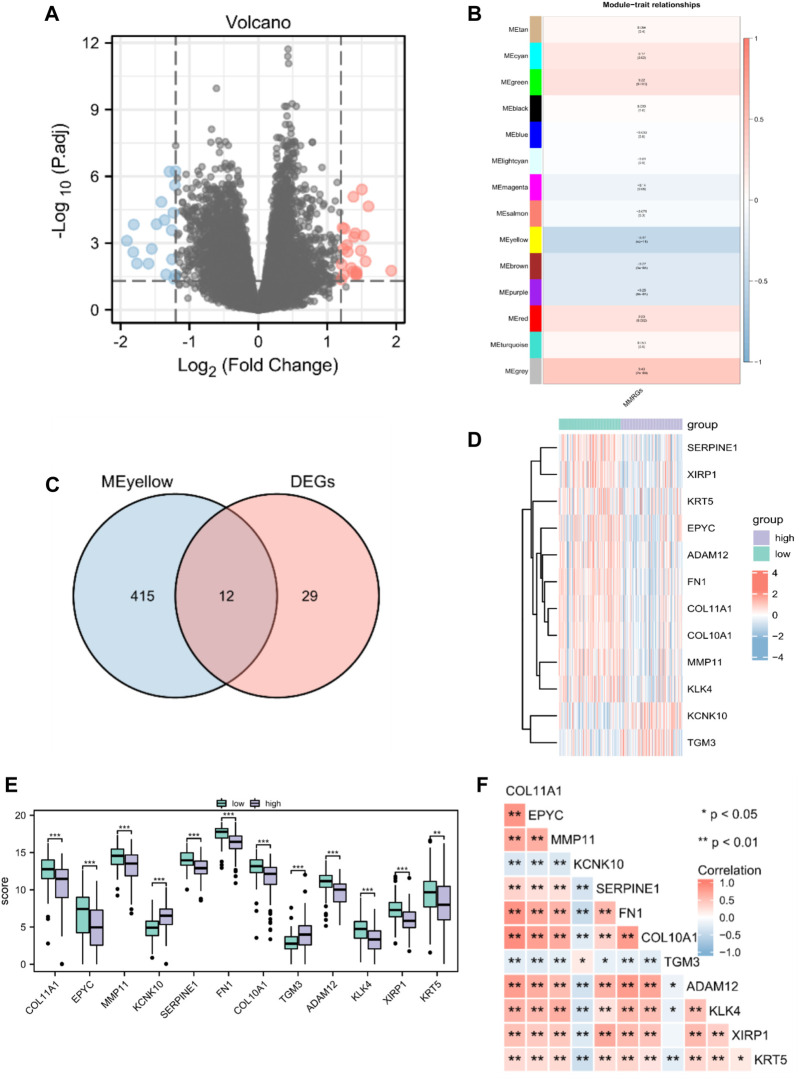
Identification of prognostic MMRGs in TCGA database. **(A)** The volcano plot of differentially expressed MMRGs in TCGA database (23 upregulated and 18 downregulated genes, |logFC| > 1.2 and *p* < 0.05). **(B)** WGCNA indicated that MEyellow modules were closely associated with the MMs. **(C)** Venn diagrams of the overlapping MMRGs between DEGs and module genes of MEyellow in TCGA-PAAD dataset. **(D)** Heatmap showing the expression of the 12 key genes between MMs-high (n = 89) and MMs-low groups (n = 89). **(E)** Differential expression of key genes between MMs-high (n = 89) and MMs-low groups (n = 89). **(F)** Heatmap showing the correlations between the 12 key genes. *, *p* < 0.05; **, *p* < 0.01; ***, *p* < 0.001.

Altogether, 226 MMRGs obtained from the GeneCards and KEGG pathway databases were enrolled after removing duplicates. By comparing the MMRG expression between high- and low-MMs groups, we identified 41 genes as differentially expressed: 23 upregulated and 18 downregulated genes (Figure 2A).

### 3.2 WGCNA

Gene modules related to mitochondrial energy metabolism were acquired via WGCNA based on TCGA database. By setting the soft threshold to 7 (Supplementary Figure S1), RsquaredCut to 0.85, and the minimum number of module genes to 25, a total of 14 non-gray modules were established. As shown in Figure 2B, MEyellow was highly related to the MMs in the non-gray module. We then took the intersection of DEGs and module genes of MEyellow in TCGA-PAAD dataset and obtained 12 key genes (COL11A1, EPYC, MMP11, KCNK10, SERPINE1, FN1, COL10A1, TGM3, ADAM12, KLK4, XIRP1, and KRT5; Figure 2C). A heatmap of the expression of the 12 key genes in the MMs-high and MMs-low groups is shown in Figure 2D. The expression levels of the 12 key genes significantly differed between the MMs-high and MMs-low groups (Figure 2E). Moreover, the constructed correlation heatmap for key genes showed significant correlations between all key genes (*p* < 0.05; Figure 2F).

### 3.3 Construction of an overall survival (OS) prognostic risk model based on MMRGs

**FIGURE 3:**
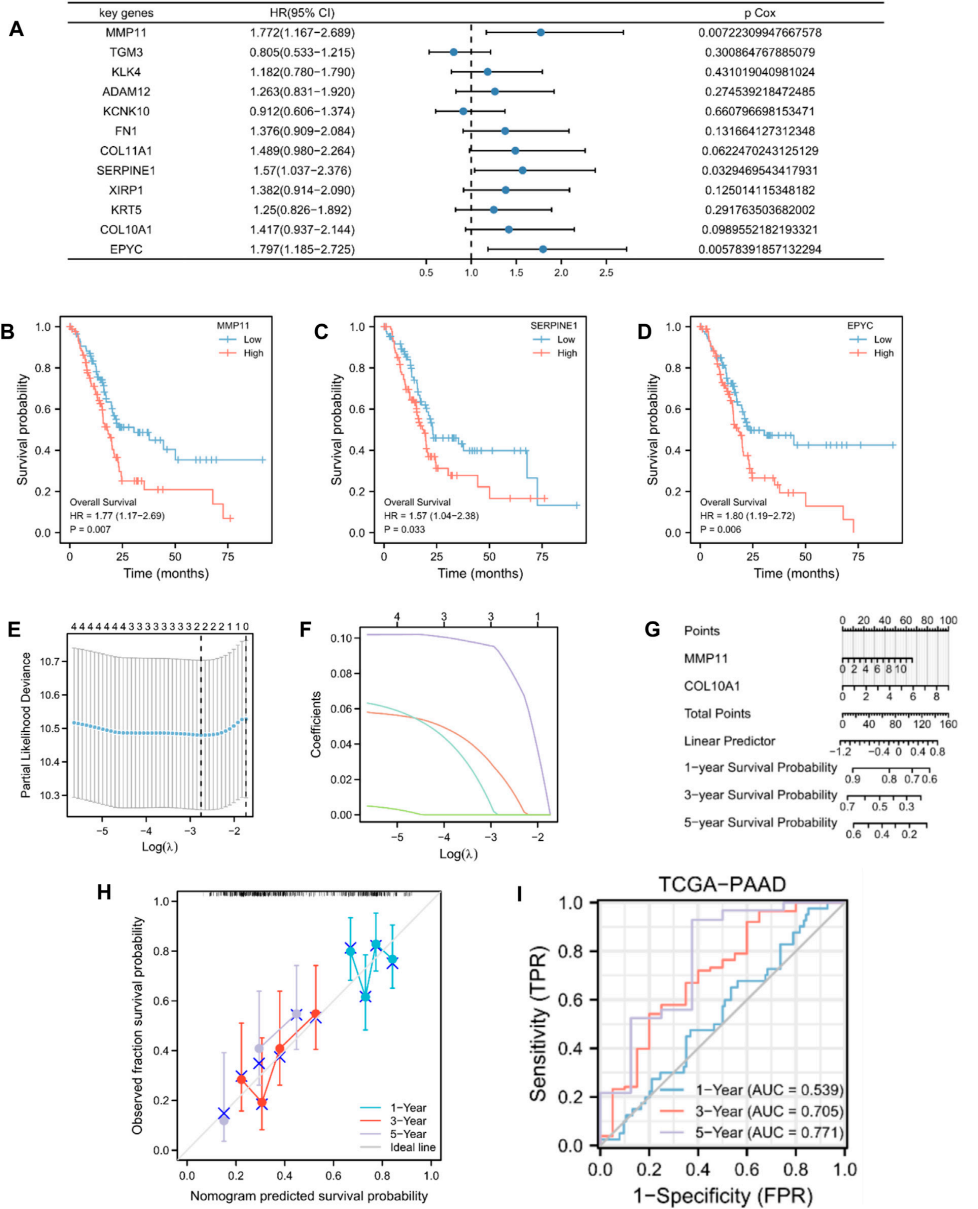
Construction and validation of MMRG-related prognostic model. **(A)** Univariate Cox regression analysis of 12 key genes. **(B–D)** Kaplan–Meier curve showing key genes (MMP11, SERPINE1, and EPYC) with *p* < 0.05. **(E)** Selection of the optimal parameter (lambda) in the LASSO model for PAAD. **(F)** LASSO coefficient profiles of the two genes (MMP11 and COL10A1) in PAAD. **(G)** Prognostic nomogram of multivariate Cox model for the two key genes. **(H)** Calibration curves in the prognostic risk model. **(I)** ROC curves and area under the curve (AUC) for 1-, 3-, and 5-year survival according to the risk model in TCGA database.

By analyzing the OS data of patients with PAAD in TCGA database, the 12 potential prognostic key genes were selected to perform a univariate Cox regression analysis. As a result, five prognostic genes (MMP11, COL10A1, SERPINE1, COL11A1, and EPYC) were identified as eligible based on the criteria (*p* < 0.1) in the PAAD datasets (Figure 3A). The Kaplan–Meier curve was plotted for the key genes with *p* < 0.05 (Figures 3B–D). The samples with high expression of MMP11, SERPINE1, and EPYC had a worse prognosis and all were risk factors (HR > 1).

To further establish an optimal prognostic gene signature for PAAD, we performed iterative LASSO–Cox regression analysis which identified a two-gene signature comprising MMP11 and COL10A1 (Figures 3E,F). The two key genes were then subjected to multivariate Cox regression analysis (Supplementary Table S2). A prognostic nomogram of the Cox multivariate model is plotted in Figure 3G, which illustrates that high expression of MMP11 and COL10A1 reduced the survival rate of patients with PAAD. Calibration curves were then plotted to validate the predictability of the model for the survival of patients with PAAD (Figure 3H). The risk score for each patient was calculated as follows:

$$Risk\;score=\left(\text{MMP}11\times 0.0548461192552124\right)+\left(\text{COL}10A1\times 0.13554174423837\right)-0.992494070149479.$$

Patients with PAAD were classified into high- and low-risk score groups based on median values. We also evaluated the relationship between risk score and MMs (Supplementary Figure S1). However, with an increase in time, the two-gene signature appeared to yield better prediction in the ROC analysis (Figure 3I). In addition, we analyzed the data of patients with PAAD from GSE28735, GSE57495, and GSE62452 as exterior validation sets to provide an in-depth illustration of the risk model performance (Supplementary Figure S1).

### 3.4 Functional enrichment evaluation of the DEGs

**FIGURE 4:**
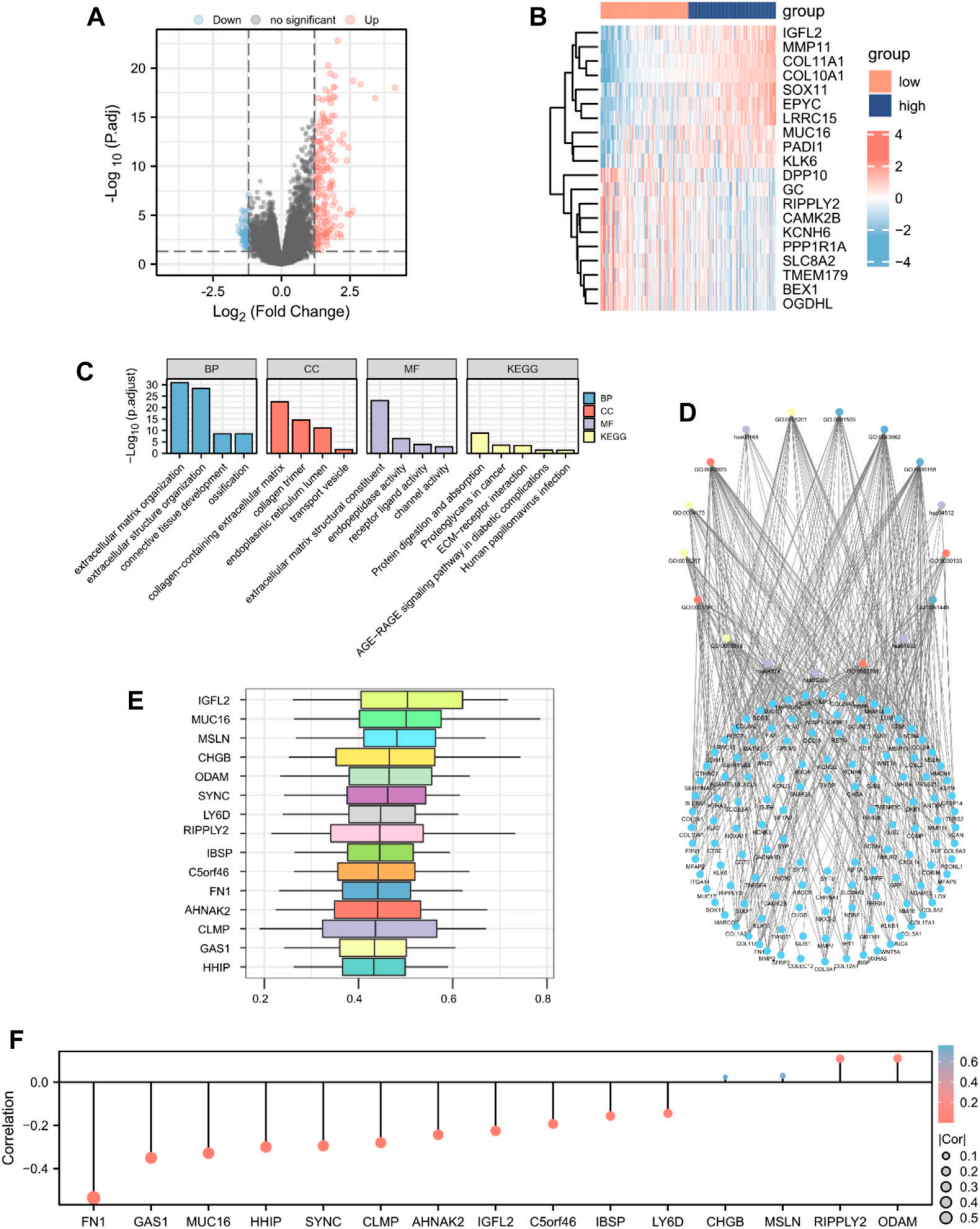
GO enrichment analysis. Identification of DEGs according to risk score-related high (n = 89) and low groups (n = 89) visualized as a **(A)** volcano plot and **(B)** heatmap (187 upregulated and 61 downregulated genes, |logFC| > 1.2 and *p* < 0.05). **(C)** Histogram and **(D)** The network diagram of GO and KEGG enrichment analysis of DEGs. **(E)** Functional similarity analysis of hub genes. **(F)** Lollipop chart of the correlation between hub genes and MMs.

To identify DEGs according to high- and low-risk score groups, we defined genes with *p*-values <0.05 and log FC > 1.2 as upregulated and genes with *p*-values <0.05 and log FC < −1.2 as downregulated. A total of 248 genes were differentially expressed; 187 upregulated genes and 61 downregulated genes (Figures 4A,B).

To determine the relationship between the prognostic risk score and GO or biological pathways, gene functional enrichment analysis was performed on DEGs between the high- and low-risk score groups in TCGA database. The top enriched GO annotations were associated with extracellular matrix organization, extracellular structure organization, and ossification in biological processes. Cellular components included connective tissue development, collagen-containing extracellular matrix, endoplasmic reticulum lumen, collagen trimer, and transport vesicle. Extracellular matrix structural constituent, endopeptidase activity, receptor ligand activity, and channel activity were the top four identified molecular functions. KEGG pathways, including protein digestion and absorption, proteoglycans in cancer, human papillomavirus infection, ECM-receptor interaction, and the AGE-RAGE signaling pathway in diabetic complications, were notably enriched (Figures 4C,D; Supplementary Table S3). We analyzed the functional similarity of DEGs using the “GOSemSim” package and selected the top 15 DEGs as hub genes (IGFL2, MUC16, MSLN, CHGB, ODAM, SYNC, LY6D, RIPPLY2, IBSP, C5orf46, FN1, AHNAK2, CLMP, GAS1, and HHIP; Figure 4E). We then plotted a lollipop chart of the correlation between the hub genes and mitochondrial energy metabolism. As shown in Figure 4F, FN1 had the highest negative correlation with MMs, whereas ODAM had the highest positive correlation with MMs. The correlation between MMs and hub genes in TCGA and GEO datasets was also validated (Supplementary Figure S2).

### 3.5 GSEA for the risk model

**FIGURE 5:**
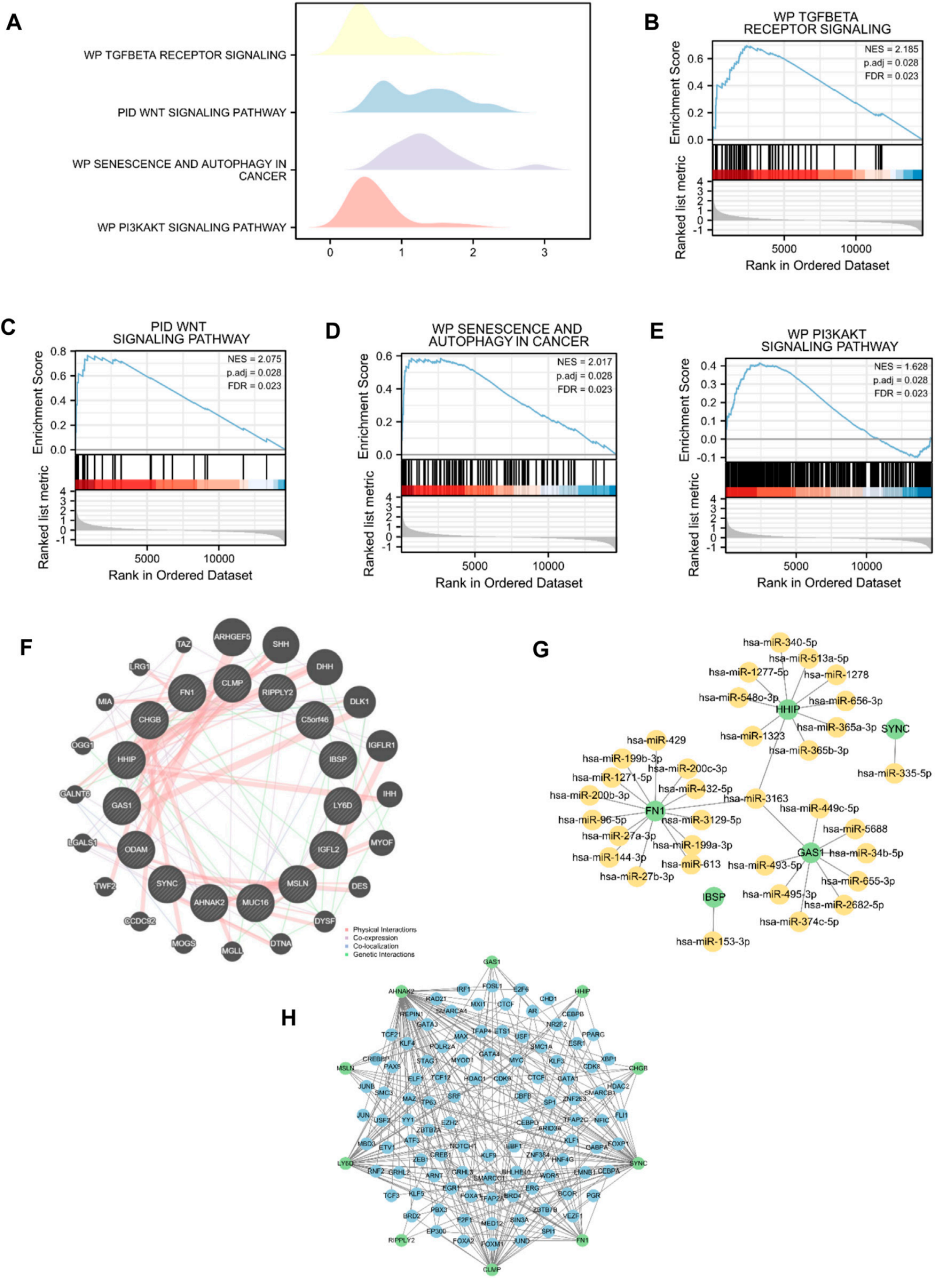
GSEA for differences in biological function between the high- (n = 89) and low-risk score groups (n = 89). **(A–E)** GSEA based on risk score-related high and low groups. **(F)** PPI network of hub genes. **(G)** mRNA–miRNA interaction network of hub genes. **(H)** Network between TFs and hub genes.

GSEA was performed to explore differences in biological function between the high- and low-risk score groups. As shown in Figures 5A–E, TGF-beta receptor signaling, the Wnt signaling pathway, senescence and autophagy in cancer, and the PI3K-Akt signaling pathway differed significantly between the two groups (Supplementary Table S4). The interaction network of functionally similar genes was illustrated according to the GeneMANIA database to observe their physical interactions, shared protein domains, gene interactions, and other information (Figure 5F). As shown in Figure 5G, the mRNA–miRNA interaction network was composed of five hub genes (HHIP, SYNC, FN1, IBSP, and GAS1) and 34 miRNA molecules, which constituted a total of 35 pairs of mRNA–miRNA interactions. According to CHIPBase and the hTFtarget database, the interaction relationship data of 10 hub genes (AHNAK2, CHGB, CLMP, FN1, GAS1, HHIP, LY6D, MSLN, RIPPLY2, and SYNC) and 101 TFs were obtained and visualized using the Cytoscape software (Figure 5H).

### 3.6 Immune-related analysis of patients with PAAD based on the prognostic risk model

**FIGURE 6:**
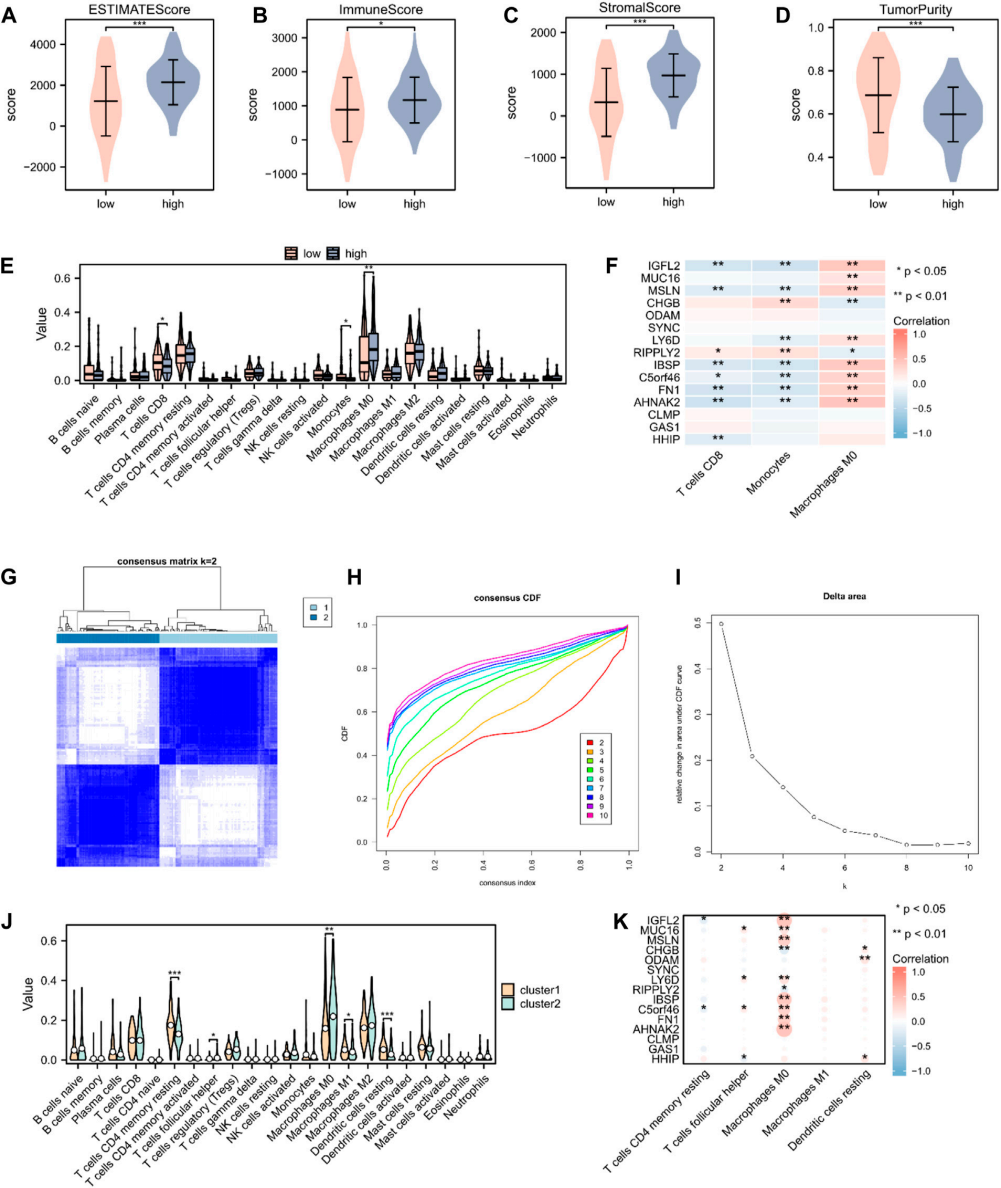
Immune cell infiltration analysis in patients with PAAD. **(A–D)** The estimate, immune, and stroma scores and tumor purity in the high- (n = 89) and low-risk score groups (n = 89) in the PAAD cohort. **(E)** Distribution of 22 immune cells in the PAAD cohort, according to CIBERSORT. **(F)** Heatmap of the relationship between infiltrating immune cells and 15 hub genes. **(G)** Heatmap of consensus clustering matrix. **(H)** Consensus cumulative distribution function diagram. **(I)** Delta area plot. **(J)** Distribution of 22 immune cells in the PAAD cohort, according to unsupervised consensus clustering. **(K)** Heatmap of the relationship between infiltrating immune cells and hub genes, according to unsupervised consensus clustering. Not significant (ns), *p* > 0.05; *, *p* < 0.05; **, *p* < 0.01; ***, *p* < 0.001.

To validate the underlying differences in immune cell infiltration between the high- and low-risk score subgroups, the estimate score (the summation of immune and stromal scores), immune score (immune cell infiltration in the tumor tissue), stromal score (substrate cells in the tumor tissue), and tumor purity were analyzed. All these scores suggested a significantly higher immune cell infiltration in the high-risk score group (*p* < 0.05; Figures 6A–D). We further explored the distinctive immune cell distribution in the high- and low-risk score groups. CD8 T cells, monocytes, and M0 macrophages significantly differed between the two groups, among which M0 macrophages had high infiltration rates in the high-risk score group, whereas CD8 T cells and monocytes had high infiltration rates in the low-risk score group (Figure 6E). A heatmap of the correlation between hub genes and immune cell infiltration is shown in Figure 6F. We then performed unsupervised consensus clustering and divided all PAAD samples into two subtypes (cluster1: n = 95; cluster2: n = 83, Figures 6G–I). Analysis of immune cell infiltration abundance revealed that CD4 memory resting T cells, follicular helper T cells, M0 macrophages, M1 macrophages, and resting dendritic cells significantly differed between the two immune characteristic subtypes (Figure 6J). A heatmap of the correlation between hub genes and immune cell infiltration is shown in Figure 6K.

### 3.7 TIDE analysis and mutation analysis of hub genes

**FIGURE 7:**
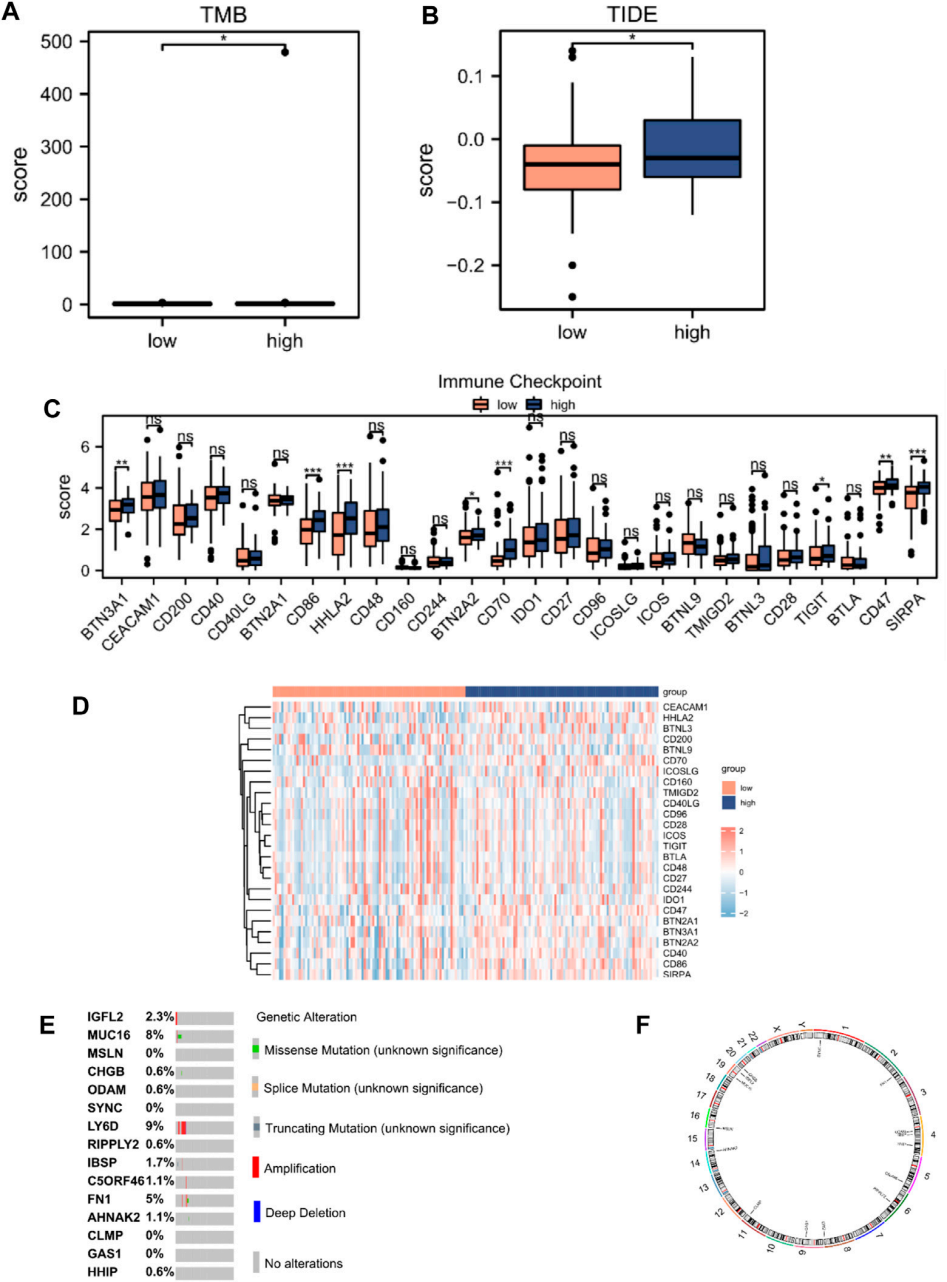
TIDE analysis and mutations of hub genes in TCGA database. **(A)** Mutation burden analysis and **(B)** TIDE score between high- (n = 89) and low-risk score groups (n = 89). **(C)** Differential expression of immune checkpoint genes according to the risk score. **(D)** Heatmap of immune checkpoint genes according to the risk score. **(E)** Mutation subtypes of hub genes. **(F)** Chromosomal localization map of hub genes. Not significant (ns), *p* > 0.05; *, *p* < 0.05; **, *p* < 0.01; ***, *p* < 0.001.

We performed tumor mutation burden analysis and observed a significant difference between the high- and low-risk score groups (*p* < 0.05; Figure 7A). We also evaluated the sensitivity of the two groups to immunotherapy using the TIDE algorithm (Figure 7B). A comparison of the immune checkpoint gene expression between the high- and low-risk score groups revealed that eight checkpoint genes, BTN3A1, CD86, HHLA2, BTN2A2, CD70, TIGIT, CD47, and SIRPA, varied between the two groups (Figures 7C,D). We analyzed the mutations in the 15 hub genes from the cBioPortal database in the TCGA-PAAD dataset (Figure 7E). The results revealed that genetic mutations primarily focused on missense mutations (unknown significance), splice mutations (unknown significance), truncating mutations (unknown significance), amplification, deep deletions, and no alterations. We also analyzed the positions of the 15 hub genes on human chromosomes using the “RCircos” R package (Figure 7F). The chromosomal localization map showed that hub genes were primarily distributed on chromosomes 1, 2, 4, 5, 6, 8, 9, 11, 14, 16, 19, and 20, among which, three hub genes were distributed on chromosome 4.

### 3.8 Nomogram construction

**FIGURE 8:**
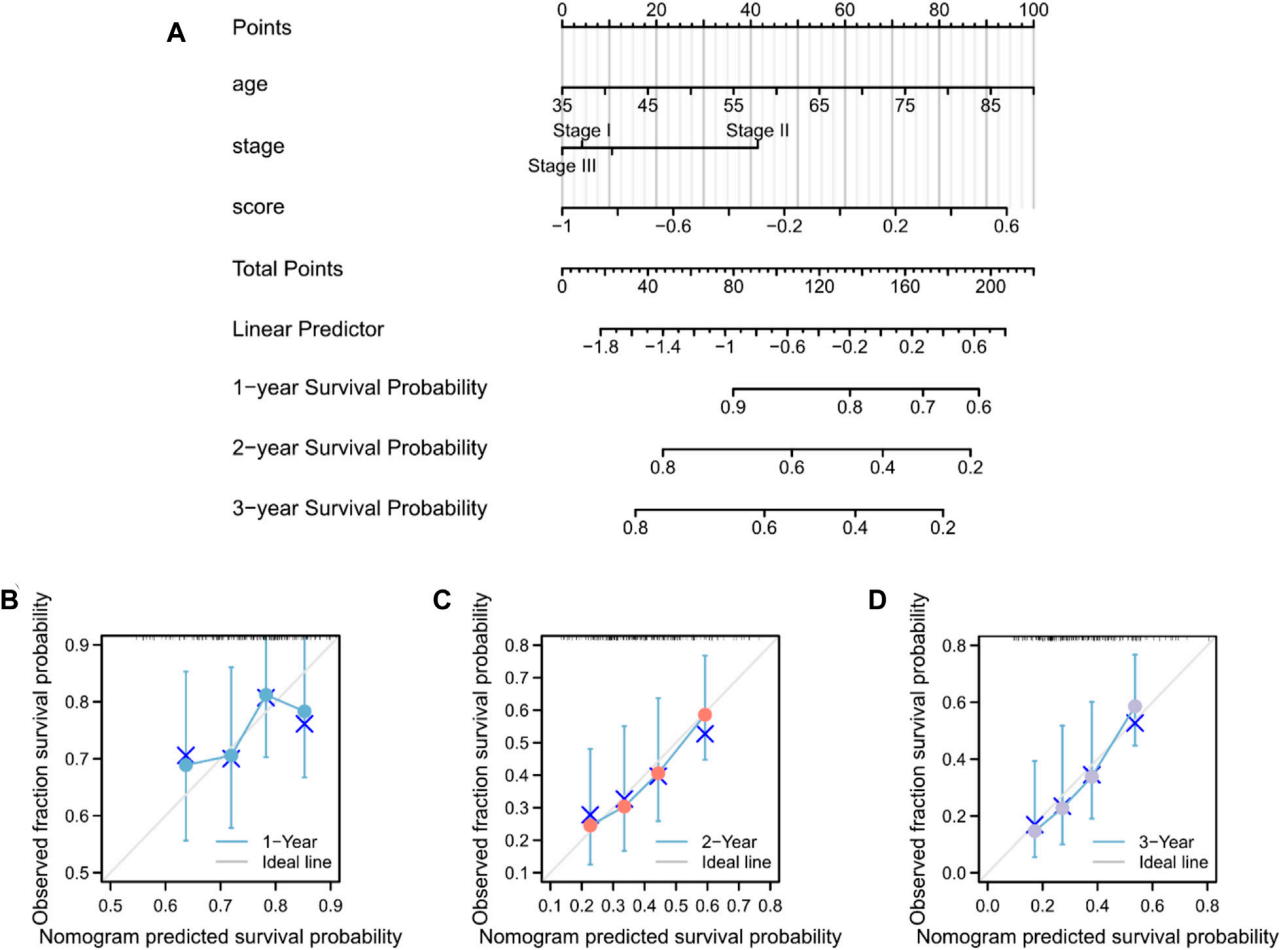
Nomogram predicting the OS in patients with PAAD. **(A)** Prognostic nomogram for the OS of patients with PAAD. Calibration curves for the **(B)** 1-, **(C)** 2-, and **(D)** 3-year OS.

The predictive nomogram was used to calculate the likelihood of patient outcome by combining clinical data and MMRG-related risk score values (Figure 8A). Figures 8B–D shows that the estimated OS rates of patients with PAAD at one, two, and three years matched well with the actual OS rates.

### 3.9 Validation of key genes *in vitro*

**FIGURE 9:**
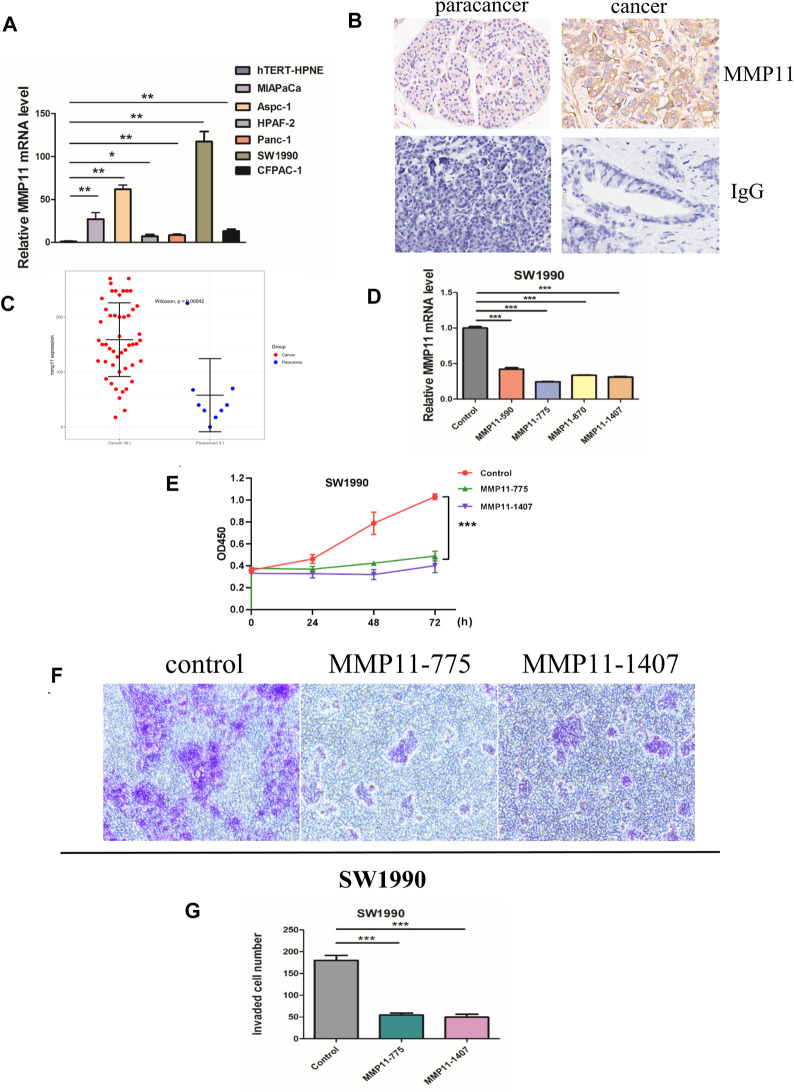
*In vitro* experiments. **(A)** The mRNA expression of MMP11 in pancreatic cancer cell lines. **(B)** Immunohistochemical analysis of MMP11 expression in PC tissues. **(C)** Statistical analysis of MMP11 expression between PC tissues and paracancer tissues. **(D)** qRT-PCR was performed to detect the efficiency of si-MMP11 transfection. **(E)** Cell viability according to CCK-8 assay. **(F)** The invasion capacity of SW1990 cell lines according to Transwell assay. **(G)** Statistical analysis of invaded cells. *, *p* < 0.05; **, *p* < 0.01; ***, *p* < 0.001.

The expression of five key genes (MMP11, COL10A1, SERPINE1, COL11A1, and EPYC) in normal pancreatic and pancreatic cancer cell lines were detected using qPCR. The results showed that MMP11 was highly expressed in pancreatic cancer cell lines, including MIAPaCa, Aspc-1, HPAF-2, Panc-1, SW 1990, and CFPAC-1 and we selected SW1990 cell lines in the subsequent cell experiments (Figure 9A). Immunohistochemistry assays showed that MMP11 expression level was significantly enhanced in PC tissues compared with in para-carcinoma tissues (Figures 9B,C). We used qRT-PCR to evaluate mRNA level of MMP11 24 h after transfection in SW1990 cell lines. The results showed that MMP11–775 and MMP11–1407 had a better knockdown efficiency, which were used in subsequent experiments (Figure 9D). CCK8 assay further showed that knockdown of MMP11 showed a significant reduction in cell viability and proliferation (Figure 9E). After siRNA knockdown, the percentage of cells crossing the plate decreased dramatically, which indicated that the invasion capacity of SW1990 cells decreased significantly, compared with the negative control group (Figures 9F,G).

## 4 Discussion

The prognosis of PAAD is extremely poor because of its late diagnosis and resistance to chemotherapy (Wu et al., 2022). Patients with PAAD who qualify for surgical resection account for less than 20% because most tumors are already at advanced stages upon diagnosis (Lindgaard et al., 2022). Thus, advanced prognostic biomarkers are urgently required to predict the survival rate of patients with PAAD.

Reprogrammed mitochondrial energy metabolism, including the tricarboxylic acid (TCA) cycle, is considered a hallmark of cancer (Di Gregorio et al., 2022; Sawant Dessai et al., 2022). Tumor cells use glycolysis and the TCA cycle to generate ATP, NADPH, and macromolecules (nucleotides, lipids, and amino acids) to synthesize substrates essential for cell proliferation (DeBerardinis and Cheng, 2010; DeBerardinis and Chandel, 2020; Ghosh et al., 2020; Vasan et al., 2020). Therefore, inhibition of the mitochondrial TCA cycle has attracted much attention in cancer treatment. CPI-613, a lipoate analog that inhibits pyruvate dehydrogenase and α-ketoglutarate dehydrogenase, showed efficacy in tumor therapy (Stuart et al., 2014; Reddy et al., 2022). CPI-613 is currently undergoing phase III clinical trials for patients with metastatic pancreatic adenocarcinoma (NCT03504423). In addition, mitochondrial energy metabolism is critically associated with antitumor immunity, and recent studies have shown that inhibiting mitochondrial transaminase regulates the immune microenvironment of pancreatic cancer to suppress tumor progression (Abrego et al., 2022). Therefore, our aim was to investigate the relationship between genes associated with mitochondrial energy metabolism and PAAD prognosis, formulating a gene-signature risk model as a biomarker for guiding the diagnosis and treatment of pancreatic cancer.

In the present study, public RNA-sequencing expression and clinical data were obtained from TCGA and GEO databases, and a total of 41 MMRGs were identified as differentially expressed, including 18 downregulated and 23 upregulated genes between the MMs-high and MMs-low groups after ssGSEA. Based on univariate Cox regression analysis, five prognostic key genes (MMP11, COL10A1, SERPINE1, COL11A1, and EPYC) were identified. Furthermore, iterative LASSO–Cox regression analysis was performed to construct a two-gene signature as a risk model for prognosis. After verification of the GEO datasets, the risk model—comprising MMP11 and COL10A1—showed good performance in predicting PAAD prognosis.

Matrix Metalloproteinase 11 (MMP11) is a member of the MMP family, which is involved in the breakdown of the extracellular matrix in normal physiological processes. The enzyme encoded by MMP11 is activated intracellularly by furin within the constitutive secretory pathway and plays an important role in the progression of epithelial malignancies. MMP11 was highly expressed in the cancerous ductal epithelium and might act as a tumor promoter in PDAC, stimulating cyclin-dependent kinase 4 and cyclin D1 (Zhang X. et al., 2020). The presence of MMP11 could exacerbate endoplasmic reticulum stress, alter the mitochondrial unfold protein response, which mediated cancer progression (Tan et al., 2020). In addition, overexpression of MMP11 provoked mitochondrial defects, which triggered aerobic glycolysis, revealing a major role in controlling energy metabolism (Dali-Youcef et al., 2016). Type X collagen α 1 chain (COL10A1), a member of the collagen family, is a gene associated with the progression of a variety of human tumors. COL10A1 regulates PDAC cell proliferation and MEK/ERK signaling pathways by binding to discoid protein domain receptor 2 (DDR2) to promote migration, invasion, and epithelial–mesenchymal transition (EMT) (Wen et al., 2022). Collagen XI, another member of the collagen family, is present in the extracellular matrix. The COL11A1 gene is involved in tumorigenesis and the development of many human malignancies. COL11A1 plays a critical role in PAAD progression by stimulating the Akt/GSK-3β/Snail signaling pathway (Wang et al., 2022) and is the key to PAAD development. In addition, COL11A1 mediates mitochondrial apoptotic evasion to enhance chemotherapy tolerance in pancreatic cancer cells (Wang et al., 2021). Circ-000510 could modulate COL11A1 expression, activating EMT in PAAD (Ma et al., 2021). Serpin Family E Member 1 (SERPINE1), as primary inhibitor of tissue-type plasminogen activator (PLAT), is involved in the regulation of cell adhesion and spreading (Planus et al., 1997), and cellular and replicative senescence (Kortlever et al., 2006). SERPINE1 was identified as a key target of the TP53/miR34a axis, with relevance to the biology of PDAC, and might serve as a potential biomarker for early detection of PDAC (Akula et al., 2020). EPYC is a member of the small leucine-rich repeat proteoglycan family, which is composed of seven exons. However, the association between EPYC and cancer has rarely been studied. To explore the expression levels of the five key genes, qPCR-based cDNA array analysis was performed. The results showed that the mRNA expression of MMP11 was significantly upregulated in pancreatic cancer cell lines than in a normal pancreatic duct epithelial cell line, which is consistent with the results of previous studies. In addition, IHC results further confirmed elevated expression of MMP11 in pancreatic cancer tissues compared with adjacent tissues. Subsequently, CCK-8 and transwell assay demonstrated that knockdown of MMP11 significantly reduced cell proliferation and invasion of SW1990 cell lines *in vitro*, suggesting MMP11 might provide new therapeutic target for the treatment of pancreatic cancer.

GO and KEGG analyses illustrated that DEGs based on the risk score were closely related to extracellular matrix organization, collagen-containing extracellular matrix, extracellular matrix structural constituent, and protein digestion and absorption. In addition, we identified 15 hub genes through the functional similarity of DEGs between the high- and low-risk score groups, among which FN1 had the highest negative correlation with MMs, whereas ODAM had the highest positive correlation with MMs. FN1 encodes fibronectin, which is involved in cell adhesion and migration processes (Owens and Baralle, 1986). Xavier et al. (Xavier et al., 2021) found that FN1 was expressed in the stroma and identified FN1 as a potential target for pharmacological inhibition in PDAC. However, the effect of FN1 on mitochondrial energy metabolism has rarely been demonstrated. Odontogenic, Ameloblast-Associated (ODAM) is frequently upregulated in hepatocellular carcinoma, colorectal adenocarcinoma, and hepatoblastoma. Yamaguchi et al. (Yamaguchi et al., 2023) identified ODAM as a novel target gene of Wnt/ß-catenin signaling, which played an important role in the regulation of the cell cycle, DNA synthesis, and cell proliferation. ODAM inhibits colorectal cancer growth by promoting PTEN and suppressing the PI3K/AKT pathway (Yu et al., 2016). Its role in pancreatic cancer has not yet been reported. GSEA revealed that the high- and low-risk score groups showed significant differences in TGF-beta receptor signaling, the Wnt signaling pathway, senescence and autophagy in cancer, and the PI3K-Akt signaling pathway, which might account for the poor PAAD prognosis owing to risk modifiers (Aguilera and Dawson, 2021; Cortesi et al., 2021; Yang et al., 2021; Stanciu et al., 2022; Yamamoto et al., 2022).

PAAD is regarded as having low immunogenicity and to lack effective immunotherapy responses (Macherla et al., 2018). The immune-related analysis was aimed at exploring promising targets for PAAD immunotherapy. The ESTIMATE analysis showed higher estimate, immune, and stromal scores in the high-risk score group, which might benefit from immunotherapy. In addition, the high-risk score group was associated with a higher infiltration of cancer-associated M0 macrophages. The relatively lower expression of monocytes and CD8 T cells in the high-risk score group indicates that monocyte and CD8 T cell function might have been suppressed.

The TIDE analysis showed higher TIDE scores in the high-risk score group, which implied a likelihood of immune surveillance escape and tumor insensitivity. Immune checkpoint analysis suggested that eight genes, BTN3A1, CD86, HHLA2, BTN2A2, CD70, TIGIT, CD47, and SIRPA, showed higher expression in the high-risk score group, which provide approaches for immune blockade therapy based on our risk model. However, considering the unsatisfactory effect of immunotherapy in recent studies and the associated controversies (Macherla et al., 2018; Leinwand and Miller, 2020; Wu et al., 2020; Chen et al., 2021), immunological function in patients with PAAD needs further investigation. Moreover, a nomogram combining clinicopathological characteristics and the two-gene signature associated with mitochondrial energy metabolism efficiently predicted PAAD prognosis at 1, 2, and 3 years.

Our study has limitations; in that, it was based on the bioinformatic analysis of two databases and validation of biological behavior *in vitro*. At the same time, we validated clinical significance of MMP11 based on PC tissue microarrays. However, further *in-vivo* experiments and specific mechanism are required. Few related studies have been published, and the role of our two-gene signature in PAAD is still vague. Therefore, its function in PAAD requires further investigation. The number of PAAD samples in TCGA database was not large enough, and the statistical significance requires further verification. Moreover, the exact molecular mechanisms underlying functional analysis and immune infiltration based on the risk model in pancreatic cancer require in-depth exploration.

## 5 Conclusion

In this study, we successfully identified an effective prognostic two-gene signature, which demonstrated an accurate prediction of survival in patients with PAAD in TCGA and GEO databases. A nomogram combining clinical characteristics and risk scores was established to evaluate the survival probability, which showed a certain accuracy. We demonstrated a close relationship between mitochondrial energy metabolism and PAAD prognosis, and the established risk model provides new prognostic biomarkers for pancreatic cancer.

## Data Availability

The datasets presented in this study can be found in online repositories. The names of the repository/repositories and accession number(s) can be found in the article/Supplementary Material.
