# Supplementary material for: Mitochondrial energy metabolism-related gene signature as a prognostic indicator for pancreatic adenocarcinoma
Source: Front Pharmacol. 2024 Mar 20;15:1332042. doi: 10.3389/fphar.2024.1332042 (PMC10987750; doi:10.3389/fphar.2024.1332042)
Supplement: Supplementary file 1 [file DataSheet1.pdf]

Supplementary Material

1 Supplementary Figures and Tables

1.1 Supplementary Figures

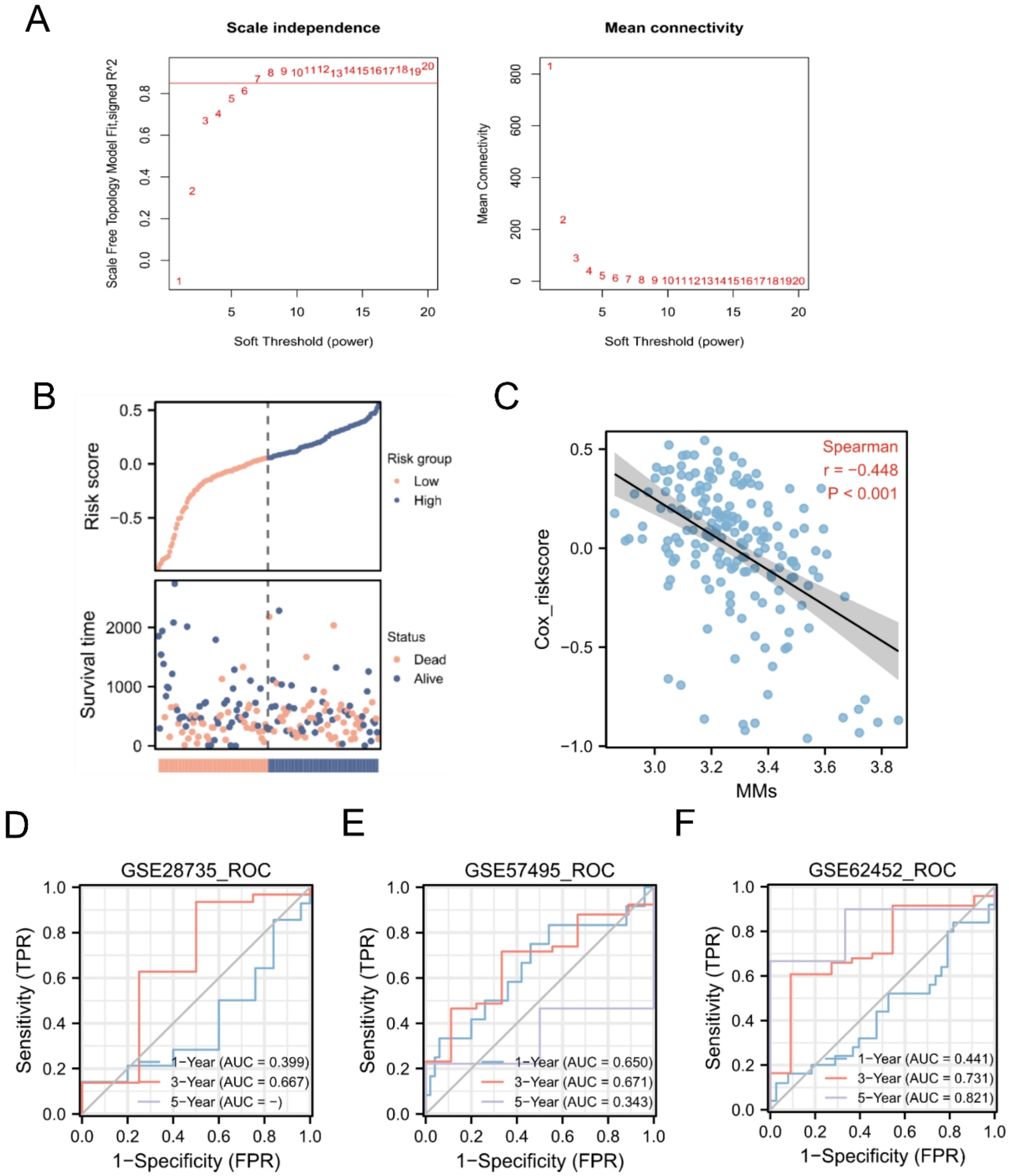

Supplementary Figure 1. (A) Soft threshold of WGCNA. (B) Risk factor diagram of the risk model. (C) Scatter plot of the correlation between risk scores and mitochondrial energy metabolism score

(MMs). (D–F) Receiver operating characteristic (ROC) curves and area under the curve (AUC) for 1-, 3-, and 5-year survival in GEO datasets of the risk model.

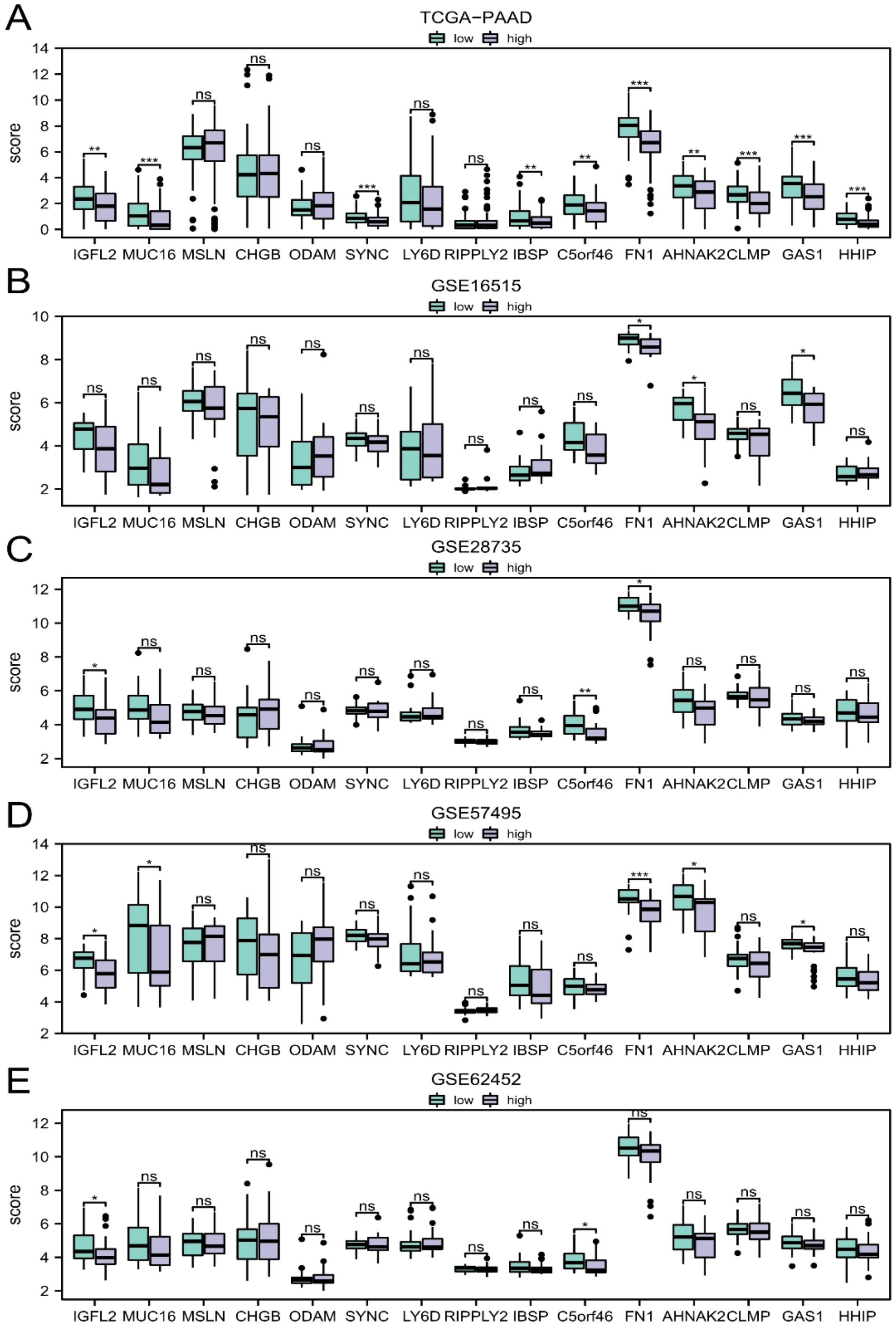

Supplementary Figure 2. (A–E) Expression of 15 hub genes in the MMs-high and MMs-low groups in TCGA and GEO datasets. Not significant (ns), $p > 0.05$; *, $p < 0.05$; **, $p < 0.01$; ***, $p < 0.001$.

1.2 Supplementary Tables

Supplementary Table 1. Characteristics of patients with PAAD in the TCGA dataset

Characteristic	Level	Overall [n (%)]
Total		178
T stage	T1	7 (4)
	T2	24 (13.6)
	T3	142 (80.7)
	T4	3 (1.7)
N stage	N0	50 (28.9)
	N1	123 (71.1)
M stage	M0	79 (94)
	M1	5 (6)
Gender	Female	80 (44.9)
	Male	98 (55.1)
Age	≤65	93 (52.2)
	>65	85 (47.8)

OS event	Alive	86 (48.3)
	Dead	92 (51.7)
DSS event	Alive	100 (58.1)
	Dead	72 (41.9)
PFI event	Alive	74 (41.6)
	Dead	104 (58.4)

DSS: disease-specific survival; OS: overall survival; PAAD: pancreatic ductal adenocarcinoma; PFI: progression-free interval; TCGA: The Cancer Genome Atlas.

Supplementary Table 2. Cox regression analysis of TCGA-PAAD dataset.

Characteristics	Total (N)	Univariate analysis		Multivariate analysis	
		Hazard ratio (95% CI)	p-value	Hazard ratio (95% CI)	p-value
MMP11	178	1.161 (1.043–1.292)	0.006	1.056 (0.897–1.245)	0.512
COL10A1	178	1.197 (1.060–1.353)	0.004	1.145 (0.955–1.373)	0.144

CI: confidence interval.

Supplementary Table 3. GO and KEGG enrichment analysis results of DEGs.

ONTOLOGY	ID	Description	Gene Ratio	Bg Ratio	p-value	P.adjust	Q value
BP	GO:0030198	Extracellular matrix organization	48/232	368/18670	3.92862E-35	1.11141E-31	9.28394E-32
BP	GO:0043062	Extracellular structure organization	48/232	422/18670	2.52345E-32	3.56942E-29	2.98165E-29
BP	GO:0001503	Ossification	26/232	398/18670	5.22121E-12	2.84034E-09	2.37263E-09
BP	GO:0061448	Connective tissue development	22/232	273/18670	4.13912E-12	2.84034E-09	2.37263E-09
CC	GO:0062023	Collagen-containing extracellular matrix	41/242	406/19717	1.04834E-25	2.91439E-23	2.39463E-23
CC	GO:0005788	Endoplasmic reticulum lumen	25/242	309/19717	9.52135E-14	8.82312E-12	7.24959E-12
CC	GO:0005581	Collagen trimer	18/242	87/19717	1.91815E-17	2.66623E-15	2.19073E-15
CC	GO:0030133	Transport vesicle	13/242	392/19717	0.001169861	0.021681416	0.017814719
MF	GO:0005201	Extracellular matrix structural constituent	30/225	163/17697	2.20841E-26	8.34781E-24	6.97394E-24
MF	GO:0004175	Endopeptidase activity	23/225	427/17697	6.01921E-09	3.25037E-07	2.71543E-07

MF	GO:0048018	Receptor ligand activity	20/225	482/17697	3.74206E-06	0.000143398	0.000119798
MF	GO:0015267	Channel activity	17/225	456/17697	7.79221E-05	0.001440555	0.001203471
KEGG	hsa04974	Protein digestion and absorption	14/94	103/8076	9.17584E-12	1.31215E-09	1.24598E-09
KEGG	hsa05205	Proteoglycans in cancer	12/94	205/8076	3.98294E-06	0.00028478	0.000270421
KEGG	hsa05165	Human papillomavirus infection	11/94	331/8076	0.001525556	0.04363091	0.041430897
KEGG	hsa04512	ECM-receptor interaction	8/94	88/8076	7.47289E-06	0.000356208	0.000338247
KEGG	hsa04933	AGE-RAGE signaling pathway in diabetic complications	6/94	100/8076	0.001048248	0.037474876	0.03558527

BP: biological process; CC: cellular component; DEGs: differentially expressed genes; GO: Gene Ontology; KEGG: Kyoto Encyclopedia of Genes and Genomes; MF: molecular function.

Supplementary Table 4. GSEA analysis of the TCGA-PAAD cohort.

ID	Set Size	Enrichment Score	NES	P value	P.adjust	Q value	rank
REACTOME_EXTRACELLULAR_MATRIX_ORGANIZATION	271	0.6996039	2.708424607	0.001282051	0.028289963	0.023074415	1812

REACTOME_COLLAGEN_FORMATION	81	0.776466957	2.607194581	0.00148368	0.028289963	0.023074415	1936
REACTOME_DEGRADATION_OF_THE_EXTRACELLULAR_MATRIX	124	0.72307795	2.561438545	0.001410437	0.028289963	0.023074415	1784
REACTOME_ASSEMBLY_OF_COLLAGEN_FIBRILS_AND_OTHER_MULTIMERIC_STRUCTURES	59	0.794643149	2.54224003	0.001533742	0.028289963	0.023074415	1726
REACTOME_COLLAGEN_DEGRADATION	60	0.783126383	2.513513756	0.001540832	0.028289963	0.023074415	1784
NABA_CORE_MATRISOME	247	0.648546315	2.484696023	0.00130719	0.028289963	0.023074415	1307
REACTOME_COLLAGEN_BIOSYNTHESIS_AND_MODIFYING_ENZYMES	60	0.764992806	2.455312429	0.001540832	0.028289963	0.023074415	1794
REACTOME_ECM_PROTEOGLYCANS	73	0.719880537	2.40294787	0.001472754	0.028289963	0.023074415	1812
REACTOME_ELASTIC_FIBRE_FORMATION	43	0.794698531	2.399688782	0.001633987	0.028289963	0.023074415	1307
PID_SYNDECAN_1_PATHWAY	46	0.78060804	2.393754976	0.001633987	0.028289963	0.023074415	1380
PID_AVB3_INTEGRIN_PATHWAY	71	0.723807549	2.387342597	0.001501502	0.028289963	0.023074415	1389
WP_TGFBETA_RECEPTOR_SIGNALING	51	0.696274413	2.185275898	0.001560062	0.028289963	0.023074415	2375

PID_WNT_SIGNALING_PATHWAY	25	0.764768679	2.075432588	0.001776199	0.028289963	0.023074415	836
WP_SENESCENCE_AND_AUTOPHAGY_IN_CANCER	96	0.585718804	2.01652725	0.001447178	0.028289963	0.023074415	663
WP_PI3KAKT_SIGNALING_PATHWAY	311	0.415328265	1.627571514	0.001243781	0.028289963	0.023074415	2400

GSEA: Gene Set Enrichment Analysis.
